# Perfusion drugs for non‑muscle invasive bladder cancer (Review)

**DOI:** 10.3892/ol.2024.14400

**Published:** 2024-04-15

**Authors:** Jingyuan Qian, Qiuchen Zhang, Yang Cao, Xi Chu, Yiyang Gao, Haifei Xu, Hongzhou Cai, Jiajia Wu

**Affiliations:** 1 Department of Nursing, Jiangsu Cancer Hospital and The Affiliated Cancer Hospital of Nanjing Medical University and Jiangsu Institute of Cancer Research, Nanjing, Jiangsu 210009, P.R. China; 2 Department of Urology, Jiangsu Cancer Hospital and The Affiliated Cancer Hospital of Nanjing Medical University and Jiangsu Institute of Cancer Research, Nanjing, Jiangsu 210009, P.R. China; 3 Department of Urology, Nantong Tumor Hospital, Nantong, Jiangsu 226006, P.R. China

**Keywords:** bladder cancer, perfusion therapy, Bacillus Calmette-Guérin, chemotherapy, interferon

## Abstract

The high recurrence rate and poor prognosis of non-muscle invasive bladder cancer (BC) are challenges that need to be urgently addressed. Transurethral cystectomy for bladder tumors is often combined with bladder perfusion therapy, which can effectively reduce the recurrence and progression rates of BC. The present review integrated and analyzed currently available bladder perfusion drugs, mainly including chemotherapeutic agents, immunotherapeutic agents and other adjuvant perfusion drugs. Bacillus Calmette-Guerin (BCG) perfusion was the pioneering immunotherapy for early BC and still ranks high in the selection of perfusion drugs. However, BCG infusion has a high toxicity profile and has been shown to be ineffective in some patients. Due to the limitations of BCG, new bladder perfusion drugs are constantly being developed. Immunotherapeutic agents have opened a whole new chapter in the selection of therapeutic agents for bladder perfusion. The present review explored the mechanism of action, clinical dosage and adverse effects of a variety of bladder perfusion drugs currently in common use, described combined perfusion and compared the effects of certain drugs on BC.

## 1. Introduction

Bladder cancer (BC) is the fourth most prevalent type of cancer among men and the most prevalent urological tumor. A total of 82,290 individuals had been diagnosed with BC by 2023, with 16,710 related deaths. ~75% of patients with BC have non-muscle invasive BC (NMIBC) (1). Transurethral resection of bladder tumors (TURBT) has been shown to be an efficient and safe treatment for NMIBC, with a postoperative recurrence rate of 50–85% (2). Postoperative intravesical bladder perfusion therapy can effectively reduce the tumor recurrence rate and improve patient survival (3).

Clinically available bladder perfusion drugs are divided into three main categories, namely chemotherapeutic, immunotherapeutic and adjuvant drugs, as well as some novel and co-perfusion drugs that are undergoing clinical trials. The present review summarized the available bladder perfusion drugs.

A summary of current intravesical agents is provided in Table I. In this section, focus shall be addressed on the mechanism and characteristics of the related drugs, clinical dosage and adverse reactions.

## 2. Chemotherapeutic drugs

### N,N',N''-Triethylene thiophosphoramidite (thioTEPA)

thioTEPA is a trifunctional alkylating agent with broad-spectrum antitumor activity. The cellular targets of alkylating agents are nucleic acids, DNA and RNA, but may also involve other cellular components (such as proteins). Alkylating agents can react with DNA in several different ways, thereby interfering with the proliferation and division of cancer cells (4,5). It is one of the most effective antitumor drugs in high-dose therapy, including superficial BC (6). The alkylating agent has been revealed to be an effective antitumor agent in several different ways, including in the treatment of BC.

In clinical practice, thioTEPA has been demonstrated to be effective in reducing the recurrence of low-grade non-invasive BC when titrated perioperatively, with efficacy comparable to that of mitomycin-C (MMC) (7). ThioTEPA can also be used in combination with other bladder instillation drugs. A previous study has reported that bladder instillation of Bacillus Calmette-Guérin (BCG) is associated with a lower recurrence rate of BC in the group using BCG, as compared with groups undergoing instillation of other drugs (8). However, the adverse effects of BCG instillation (including difficulty in urination or hematuria) should not be underestimated, and therefore a strategy of combining thioTEPA with BCG is occasionally adopted, which ensures the therapeutic efficacy and reduces the adverse effects (9).

The main advantages of thioTEPA in clinical use are its low cost, favorable patient tolerance and moderate efficacy. ThioTEPA is known to be absorbed through the intact urinary epithelium, with absorption increasing in the presence of inflammation, tumors or trauma to the urinary epithelium (for example, surgery) (10). ThioTEPA, however, has not been widely used, primarily due to its severe myelotoxicity. Myelosuppression is a side effect caused by absorption of the drug through the bladder mucosa, and the higher the dose of thioTEPA, the higher the rate of myelosuppression. Therefore, in the absence of myelotoxic dose limitations, the dose of thioTEPA can be substantially increased, for example, in bone marrow transplant patients during the perfusion of BC to reduce the recurrence rate (6).

### Gemcitabine

Gemcitabine is a pyrimidine antimetabolite antitumor drug that may have the effect of disrupting cell replication and acts specifically in the S phase of the cell cycle (11). When gemcitabine enters the cell, it is activated by deoxycytidine kinase and converted into gemcitabine diphosphate and gemcitabine triphosphate in the cell (12). Among them, Gemcitabine diphosphate can promote the competitive binding of Gemcitabine to DNA through a synergistic effect, preventing further synthesis of DNA to exert its antitumor effect (11,13).

Gemcitabine is a relatively new antitumor drug with activity against metastatic BC. In a study of patients with NMIBC, gemcitabine was identified to have a favorable safety profile and potential as an intravesical agent for recurrent disease (14). Gemcitabine is often used clinically to treat NMIBC by bladder instillation and often in combination with drugs such as cisplatin to prevent bladder tumor recurrence (15). Compared with systemic chemotherapy, it is characterized by precise treatment, effectiveness and less systemic toxic effects (16).

Gemcitabine can also be used in thermal therapy. A recent case report indicated that gemcitabine intravesical hyperthermia combined with intravenous tislelizumab had a favorable antitumor effect on patients (16). Moreover, intravesical hyperthermic bladder perfusion has a synergistic effect with various chemotherapeutic agents in the application of BC, which can reduce the recurrence rate and the incidence of radical cystectomy compared with normal infusion chemotherapy, and has a higher safety profile (17–19).

In patients with NMIBC, 2 g gemcitabine in 50 or 100 ml of saline is often applied once a week for 6 weeks (induction therapy) or as a single infusion immediately following transurethral resection of bladder tumors (TURBT) (20). Adverse events of intravesical instillation of gemcitabine can be categorized as local and systemic; local adverse events include urinary frequency, urgency, dysuria, hematuria, bladder or pelvic pain, with prostatitis being the most common (21). As compared with the currently commonly used instillation drug BCG for intravesical therapy, gemcitabine has fewer adverse events, has similar results in intermediate-risk patients, and has improved results in BCG-refractory patients, but has poorer results in high-risk patients. Gemcitabine is more active and less toxic than filgrastim C (22).

### Epirubicin (EPI)

EPI is a chemotherapeutic anthracycline (23) that exerts its antitumor effects by interfering with DNA synthesis and function, and is most active during the S phase of the cell cycle (24). Thermotherapy, as adjuvant therapy, can be used in combination with chemotherapy regimens to induce irreversible cellular DNA damage, thus achieving therapeutic effects (25). EPI is effective in controlling the recurrence and progression of disease in intermediate and high-risk NMIBC patients with recurrence and progression over a 5-year period (26).

In the clinical management of TURBT for the treatment of NMIBC, EPI is usually used in combination with thermal bladder perfusion and exhibits strong synergistic effects. In clinical practice, EPI is usually heated to 40–43°C and instilled into the bladder. EPI 40–43°C thermotherapy disrupts DNA repair through direct cytotoxicity, causing cellular M- and S-phase cell cycle arrest, permitting a transient decrease in RNA synthesis and a prolonged decrease in DNA synthesis (27); it has immune system-enhancing effects, which can increase the number of macrophages and dendritic cells that are lost as a result of chemotherapy (28); thermotherapy also improves tumor blood flow, including improved tumor oxygenation and drug delivery (27). In addition to these thermotherapeutic effects, EPI acts between DNA base pairs in cancer cells through its own direct toxic effects, inducing DNA double-strand breaks and further interfering with their transcriptional processes (29). The direct cytotoxicity of hyperthermia combined with the cytotoxicity of EPI enhances the lethal effect on BC.

Brummelhuis *et al* (26) reported a synergistic effect of intravesical hyperthermia combined with EPI in the treatment of metastatic bladder cell carcinoma, which could reduce LD50 by 329-1,823%. This is significantly higher than the effect of mitomycin C (MMC), which reduces the LD50 by 16.7 to 483%, and gemcitabine, which reduces the LD50 by 17.4 to 50% (19,30). However, the LD50 of gemcitabine was only reduced by 17.4–50% under hyperthermia conditions, and Van Bree *et al* (31) even reported no synergistic cytotoxicity of gemcitabine under similar experimental conditions. On the other hand, epothilones, a DNA damaging agent with a markedly higher molecular weight, demonstrated a 329–1,823% reduction in LD50 under high temperature conditions. Apparently, drug-specific activation at high temperatures is more likely than an increase in drug uptake. This requires more in-depth studies in the future.

EPI is less cardiotoxic and more efficacious than the widely used adriamycin derivatives (19,23,31). However, it can still cause a variety of adverse effects, among which acute or chronic cardiotoxicity occasionally causes irreversible damage to the patient and may lead to transient arrhythmias and electrocardiographic alterations. The cardiotoxicity of EPI is ~66% of that of doxorubicin (DXR), but it remains the most serious problem in cancer treatment. In addition to this, nausea, vomiting and hair loss are common. The appearance of these side effects may occasionally require discontinuation of EPI therapy, resulting in an inadequate antitumor effect (24,32).

### Pirarubicin

Pirarubicin (4′-O-tetrahydropyramycin), a semi-synthetic anthracycline glycoside, is a chemotherapeutic agent used for intravesical therapy (33). Pirarubicin arrests the cell cycle in the G2/M phase and inhibits cancer cell proliferation in a time- and concentration-dependent manner (34). Okamura *et al* (35) reported that a drip infusion of Pirarubicin immediately after electrodesiccation of transurethral bladder tumors reduced the recurrence of superficial BCs. Local side effects produced by Pirarubicin mainly include urinary frequency, difficulty in urination and macroscopic hematuria (36).

One comparative trial compared the efficacy and safety of a novel combination of thermal chemotherapy with piroxicam and plain infusion of piroxicam in patients with intermediate- to high-risk NMIBC. The results revealed that the combination of thermal chemotherapy with piroxicam reduced the recurrence and progression rates after TURBT for BC without increasing the probability of adverse events (37). Another study showed that piroxicam had an improved thermo-synergistic effect and was more potent in eliminating tumor cells than fluorouracil, cisplatin and MMC (38). Thermal therapy reduces the structural stability of DNA and promotes the opening of the DNA strands of tumor cells, destroying their structure (39). In addition, thermal chemotherapy is also effective for the treatment of NMIBC, DNA strand opening, destruction of DNA structure (39) and increase in the binding of Pirarubicin to the DNA of tumor cells (40). However, due to the small number of samples, the specific mechanism of Pirarubicin bladder thermal perfusion combined with TURBT in the treatment of NMIBC has not yet been fully clarified, and future in-depth studies are needed.

### MMC

MMC, an antitumor antibiotic isolated from the fermentation of *Streptomyces*-dissecting filtrate, is a cell cycle non-specific drug that cannot be absorbed by the mucous membranes (41). MMC is usually used after surgery for superficial BC to prevent tumor recurrence, with favorable short-term efficacy; its long-term efficacy is yet to be observed. The recommended regimen for the instillation of MMC in clinical practice is 20–40 mg + 30 ml of 0.9% sodium chloride injection, and patients are required to change their position every 0.5 h (for example, from supine position to right or left lateral position) (42). The antitumor mechanism of MMC is not known, but it has been identified to be effective in preventing tumor recurrence.

A recent study has reported that MMC performs hyperthermia intravesical chemotherapy at least as well as BCG in terms of safety and effectiveness (42). In the case of thermal perfusion, the increase in temperature leads to enhanced blood perfusion and increased cell permeability, thus increasing the uptake of MMC and exerting the synergistic effect of thermal therapy and chemotherapy, which can achieve a more favorable therapeutic effect. In clinical practice, the mainstay of MMC (43) thermotherapy is the infusion of 80 mg of MMC once a week for 40–60 min per cycle for 8 weeks. Thermal bladder perfusion with MMC is very safe, reliable and efficacious, provided that treatment is appropriate and adjustments are made in a timely manner. Recurrence rates have been reported to be significantly lower in patients undergoing thermal bladder perfusion with MMC than with regular perfusion (44). The use of MMC has been revealed to be effective in the treatment of BC.

The advantage of MMC over other drugs is that it is inexpensive. However, MMC poses certain challenges; it has poor thermal stability, and heating causes a decrease in its concentration, which affects its efficacy. In addition, certain patients are allergic to MMC, which is a factor for consideration (44). Due to the large molecular weight of MMC and its low mucosal absorption, it may irritate the bladder during instillation, causing short-term side effects such as gross hematuria, chemical cystitis, hepatic and renal impairment, and leukopenia. Long-term side effects have not been reported.

## 3. Immunotherapeutic drugs

### BCG

Since Morales *et al* (45) reported the prophylactic effect of the local application of live BCG to the bladder in 1976, intravesical BCG therapy has gradually become the most successful immunotherapy for BC, and is considered the standard approach for managing intermediate to high-risk NMIBC (46).

Due to the open trauma to the bladder after surgery and the tendency for immediate instillation to cause serious side effects, BCG instillation is usually started two weeks after surgery. Currently, treatment generally takes the form of a 6-week infusion to induce an immune response, and cystoscopy followed by several more weeks of infusion for reinforcement, which allows the patient to receive an enhanced immune effect (45,46). The BCG dose and treatment regimen required to initiate a therapeutic response vary among patients, and the optimal schedule and duration are still being tested (47,48).

The current management of NMIBC consists of initial complete resection of TURBT and postoperative monitoring with cystoscopy. Cancer recurs in 50–70% of patients with NMIBC who do not undergo adjuvant post-resection therapy, especially those with high-risk tumors (47). Adjuvant intravesical BCG is the most effective treatment to reduce the risk of recurrence in patients with intermediate- and high-risk NMIBC (49). An early study demonstrated a 5-year recurrence-free survival (RFS) of 80% in patients who received intravesical BCG and transdermal vaccination, compared with 48% in patients who did not receive BCG (50).

The antitumor mechanism of BCG is also associated with the local immune response (49). Following perfusion, BCG binds to urothelial cells via fibronectin and integrin receptors, leading to a sarcoma-free inflammatory response with infiltration of granulocytes, macrophages and lymphocytes (51). This leads to the induction of chemokines (cytokines including IL-1, IL2, IL-6, IL-8, IL-12, IL-18, IFN-γ, TNF-α and granulocyte-macrophage colony-stimulating factor) (48). Soluble intercellular adhesion molecule-1 (ICAM-1) has recently been found in urine following BCG treatment, and ICAM-1 expression on tumor cells may predispose tumor cells to cell-mediated cytotoxicity (52). Furthermore, BCG induction upregulates the expression of MHC class II antigens by urothelial cells, which is associated with tumor immunity (53,54). A recent study also confirmed the ability of BCG to induce T-cell-dependent tumor-specific immunity and demonstrated that BCG-specific γδ T cells are required for the induction of conventional αβ T-cell-mediated tumor-specific immunity (55).

BCG has a direct cytotoxic effect on, among others, tumor cells *in vivo* (56). It has been reported that BCG can directly eliminate BC cells by activating TLR4, TLR7 and TLR9 proteins through the caspase 8 signaling pathway (57). BCG can activate the pro-apoptotic proteins BID and pro-caspase 9 by activating and increasing the expression of lysosomal hydrolase body protease B, which ultimately leads to apoptosis (58). The direct cytotoxic effects of BCG on BC cells may also lead to cell necrosis. Therefore, BCG is a type of prescription drug and has some toxicities (47,59,60). In addition, it has been shown that BCG action results in caspase-independent impairment of cell membrane integrity, ultrastructural changes and release of the necrosis-associated chemokine high molecular base box protein 1 (HMGB1) (61,62). HMGB1 is a useful marker of necrosis. These changes suggest that BCG causes necrosis in human BC cells.

The most common side effect of BCG is irritating urinary symptoms, such as difficulty urinating, frequency and urgency. Persistent BCG infection can also lead to several common complications, including the localized spread of BCG leading to, among others, cystitis and prostatitis, as well as spread to distant organs leading to hepatitis and pneumonia. The number of serious adverse events occurring in patients after receiving multiple infusions is rare, such as Wright's syndrome, parotid gland infections, cutaneous fistulas, pleural effusions, lumbar muscle abscesses, iliac artery ruptures and Ponce's disease (63,64).

### IFN

The IFN system consists of cells that synthesize IFN in response to an external stimulus, such as a viral infection, and by establishing an antiviral state. The IFN response represents an early defense of the host and occurs before the onset of the immune response. IFN has a wide range of biological activities in addition to the characteristics of its antiviral activity (65,66).

In IFN-stimulated fibroblasts, concomitant stress signals induce the recruitment of other p38-dependent factors to IFN-stimulated genes, thereby synergistically enhancing the expression of these genes. Activated fibroblasts may be involved in antitumor immune responses and thus act as killers of superficial BC (67,68). Because cancer-associated fibroblasts (CAFs) play an important role in the response to cancer therapy, CAFs have a large number of phenotypic and functional specificities in tumors of different tissue origins. Using single cells from patients with BC for RNA sequencing, researchers found that a characteristic CAF subpopulation, the urea transporter SLC1A1 solute carrier family 1 member 1 (SLC14A1), is expressed *in vivo* (69,70). This subpopulation is induced by IFN signaling and is active in BC cells through the WNT5A paracrine pathway. The activation of cGAS-STING signaling in tumor cells drives IFN production as a process of cGAS-STING signaling and SLC14A1 CAF differentiation. In addition, IFN inhibits SLC1A1 CAF formation by targeting STAT14 or STING, sensitizing tumor cells to chemotherapy. However, BC patients with a high percentage of SLC14A1 CAF in their tumors exhibit adverse effects independent of cancer stage and less effective neoadjuvant chemotherapy, as IFN therapy is usually used in combination with surgical treatment, conventional chemotherapy or immunotherapy (70,71).

From the aforementioned evidence, it is clear that IFN can be used for bladder perfusion therapy. In some clinical cases, IFN is often used in combination with chemotherapy and immunotherapy with significant efficacy. However, it responds poorly to combination therapy with other drugs, such as BCG (67,72). In combination therapy, the toxicity of IFN can be significant if pharmacologic doses are used, and IFN is currently administered primarily by the intravenous and intramuscular routes. Clinical results have shown that high serum IFN titers correlate with cancer treatment outcomes. In addition, since IFN is readily and rapidly filtered by the kidneys, intravenous administration usually results in substantial renal losses. In conclusion, the current need for improvement in this combination therapy is the route of administration. Its efficacy may be greatly improved if a new route of administration, the lymphatic route, is used. The basic strategy is to transfer most of the IFN to the lymphatic pool, minimizing direct absorption into the blood. That way, the IFN concentration ratio in lymph or plasma will be >1 and similar to that observed during physiological response. If the therapeutic indexes are comparable in subsequent trials, lymphatic route administration may become the preferred option for combination tumor therapy. In a study on adjuvant IFN therapy for BC, urine was collected and tested immediately after 7 days of treatment of patients receiving IFN-adjuvant chemotherapy (73). Urinary elastase, serum and urinary neopterin concentrations were measured, and a cytologic evaluation of urine smears and CD68 repainting of bladder tumors were performed. The results indicated that urine contains a large number of immunomodulatory molecules, and in patients there is clear evidence that urine excreted immediately after MAK infusion contains high levels of IL-8, molecules that may profoundly alter the nature of cytokines produced by fibroblasts such as CAFs and play an important role in the treatment outcome.

However, currently used IFN, both purified natural IFN and IFN produced by recombinant DNA technology, have numerous toxicities and often lead to leukopenia, anemia, headache, fever, liver function abnormalities and central nervous system toxicity when used clinically. Clinically used IFN inducers, such as polymyxin, are highly toxic and expensive, and ribonuclease, which destroys polymyxin, exists in human serum, making it difficult to promote the use of IFN in the clinical practice.

### Hemocyanin (KLH)

KLH, extracted from the hemolymph of *Megathura crenulata* (keyhole limpet), is a multifunctional protein that not only functions as an oxygen transporter, but also stores energy substances, maintains osmolality, possesses phenol-oxidase activity, and has antimicrobial properties, making it an important immune molecule in arthropods and mollusks. As a potent immunogen, it was found to have the potential to be an immunotherapeutic alternative to BCG for the treatment of BC, after being tested in a mouse bladder tumor model (74).

In BC cell isolation assays, KLH exhibited antiproliferative and pro-apoptotic activities against tumor cells (75). In a mouse bladder tumor model (MBT2), the use of KLH as a carrier protein-coupled immunoreactive drug significantly reduced tumor incidence and growth, and mouse mortality, and its antitumor activity was very similar to that of BCG. In the MBT2 model, Lockjaw capsule hemocyanin was found to be both safe and effective, and is an immunomodulatory agent to consider for use in clinical trials (76).

Most adverse reactions to KLH are due to its endotoxin contamination and loss of activity upon freezing. Although high molecular weight natural KLH has been widely used (73), most easily prepared KLH is not suitable for use in human clinical trials. Due to the high endotoxin content of these products and the fact that the production of hemolymph serum is accomplished by extraction from frozen animals, precipitation may occur during cryoprocessing of KLH. In addition, it has been revealed that freeze-drying of natural KLH may result in loss of activity. Therefore, there is still a need to find a suitable method for the preparation of KLH.

### Group A streptococcus

Due to the shortcomings of bladder infusion of BCG vaccine for immunotherapy, its toxicity and side effects are high, and it is ineffective in certain populations (64). Therefore, sapylin (Group A streptococcus for injection) is often used in the treatment of superficial BC. Sapylin is an inactivated bacterial preparation that is processed by inactivating, freezing and drying group A streptococcus bacteria to completely inactivate the pathogenicity of the bacteria, leaving only their antigenic properties intact.

At present, it is considered that sapylin exerts antitumor effects through the following mechanisms: i) Mobilizing the body's cellular immune system to activate macrophages, natural killer cells, lymphokine-activated killer cells and a variety of T-cells to eliminating tumor cells; ii) stimulating the body's immune system to produce a variety of cytokines, such as TNF, IFN and IL, to participate in the antitumor effect. *In vitro*, sapylin can stimulate normal peripheral blood mononuclear cells to synthesize and secrete high concentrations of TNF, IFN and IL, and can significantly inhibit the growth of Tz and KK-47 bladder uroepithelial cell carcinoma cell lines, which is positively correlated with the concentration of sapylin; iii) increase the ability of the cellular immune system to bind with the tumor cells of the bladder to enhance the antitumor effect; iv) stimulation of peripheral blood mononuclear cells to produce cytokines enhances antitumor effects in a pattern similar to that of BCG, but with a stronger effect of sapylin (22,77,78).

Sapylin has a certain therapeutic effect on low, intermediate and high-risk NMIBC. Some patients infused with sapylin may experience significant bladder symptoms, including urinary tract irritation and hematuria, which are generally mild. A few patients may also experience immune response, including different degrees of fever and body tingling, which are normal phenomena (79–81).

In conclusion, transurethral plasma electrosurgery combined with sapylin bladder instillation is an effective treatment method, with a short recovery time after electrosurgery, and easy and feasible application of postoperative sapylin bladder instillation, which is worthy of wider use in the clinic as a treatment method for high-risk NMIBC. However, the dosage and duration of sapylin infusion need to be further studied, and the long-term efficacy remains to be observed.

### Adriamycin

Adriamycin is an anthracycline antitumor drug with a high antitumor activity and few side effects. It is a non-specific cell cycle drug with a broad antitumor spectrum; however, it is associated with certain toxic side effects on the heart and the inhibition of bone marrow growth. It has been noted in the literature that the mechanism of action of Adriamycin is also related to cellular replication and transcription, and that it has an obvious inhibitory effect on the synthesis of DNA and mRNA, which can be embedded between the base pairs of cellular genes, affecting the process of gene transcription and interfering with the formation of messenger RNA, thus preventing the replication of tumor cells and achieving an antitumor effect (82).

DXR is a type of Adriamycin. It is commonly used clinically for intravesical instillation chemotherapy. DXR is administered in doses of 30–100 mg, and the concentration range is 0.5–2 mg/ml when diluted with saline (83). Several studies have reported a role for DXR in preventing tumorigenesis following transurethral resection (84–87).

The unique advantages of Adriamycin are its high penetration, which ensures a strong eliminating effect on tumor cells with less time and number of instillations, and its specificity, which reduces the side effects to some extent (88). Adriamycin is easy and inexpensive to administer via instillation, which makes it a routinely effective method worthy of attention; it is also effective in improving the quality of life of patients and decreasing the rate of recurrence. Adriamycin can also be used in combination with IFN to improve efficacy and reduce tumor recurrence.

## 4. Adjuvant drugs

Adjuvant medications are also used to relieve certain symptoms of BC, such as silver nitrate for hemostasis, lidocaine for pain relief and hyaluronic acid for repairing the mucous membranes of irrigated cystitis. In the following section, several common adjuvant drugs for bladder perfusion were introduced.

### Sodium hyaluronate

Sodium hyaluronate, an intrinsic component of the human body, is a glucuronic acid with no species specificity, which is widely found in tissues and organs such as the placenta, amniotic fluid, crystalline lens, articular cartilage and dermal layer of the skin. It is distributed in the cytoplasm and intercellular matrix, and plays a lubricating and nourishing role for the cells contained therein and the cellular organs themselves, as well as providing a microenvironment for cellular metabolism.

As an adjunct to bladder perfusion medication, sodium hyaluronate has multiple roles. Postoperative pain after transurethral cystectomy is a major problem. Sodium hyaluronate, on the other hand, significantly reduces pain and the effect is positively correlated with the duration of treatment. Patients injected with sodium hyaluronate showed a significant decrease in pain threshold and a slight increase in maximum urine output (89,90). In addition, Takahashi *et al* (32) demonstrated that sodium hyaluronate was effective in promoting the healing of bladder mucosal epithelium and inhibiting bladder fibrosis through rabbit experiments. In conclusion, sodium hyaluronate can be used as an adjuvant drug for bladder perfusion to improve the recovery status of postoperative patients.

### Lidocaine

Lidocaine is a local anesthetic that is widely used in clinical practice for local anesthesia and analgesia, and plays an important role in the pharmacological mechanism of bladder perfusion analgesia; it also has other non-anesthetic effects (91). There is evidence that lidocaine may have antitumor effects on a wide range of cancer cells and may be sensitive to antitumor drugs. There are also results from several other retrospective studies suggesting that different anesthetic techniques affect RFS or overall survival in cancer patients, and that the mechanism may be associated with the probability that anesthetic techniques influence the immune system and the stress response associated with surgery.

In clinical practice, lidocaine is the most used local anesthetic to inhibit the proliferation of BC cells, but its downstream specific molecular mechanisms are unknown. Previous findings suggest that lidocaine affects BC cell proliferation by regulating the cell cycle and the Bax/Bcl-2 ratio. Lidocaine downregulates ICMT and inhibits BC cell proliferation (92,93). In conclusion, postoperative infusion of lidocaine in combination with other chemotherapeutic agents may be more effective than monotherapy in the treatment of BC, and further studies are necessary to explore this possibility.

## 5. Combined perfusion

The purpose of combining medications is to mitigate adverse effects during or after the completion of treatment, to enhance the effects of the infused medication, as well as to improve patient experience. Common combinations typically include BCG, lidocaine, antibiotics and sodium hyaluronate.

BCG is a combination drug commonly used to enhance the efficacy of perfusion medications. Randomized controlled trials and meta-analyses have shown that intravesical instillation of BCG reduces recurrence and prevents, or at least delays, the progression of malignancy (94). This may be associated with the action of BCG on immune cells. It has been revealed that BCG-inoculated tumor cells induce an immune response in the host through both non-specific and specific cell-mediated mechanisms, leading to the improved recognition and subsequent destruction of tumor cells (95). There is also a potential mechanism through which pre-existing high-frequency T-cell responses to BCG could enhance the efficacy of the perfused drug (96).

Lidocaine is an amide local anesthetic with multifactorial pharmacological effects, including analgesic, antimuscarinic, anti-inflammatory and antinociceptive properties (97). It can be used to effectively improve bladder irritation signs and patient experience.

Since perfusion therapy is often used after surgery, it is essential to consider the patient's risk for a urinary tract infection. Adding antibiotics to perfusion drugs can reduce the metabolic loss of antibiotics, decrease side effects and recurrence rates, lower the chance of bacterial resistance and promote the local bactericidal efficacy of antibiotics (98). However, the interactions between the two remain to be investigated. A summary of combined perfusion drugs and effects is provided in Table II.

## 6. Conclusion

At present, BCG perfusion still has a high status in the section of perfusion drugs; other drugs have no obvious efficacy in the treatment of BC, and BCG is usually selected for bladder instillation to treat BC in clinical practice. However, the BCG vaccine has strong side effects, therefore its clinical application is very limited, and there is an urgent need to find a drug with outstanding efficacy and low side effects to replace the BCG vaccine.

## Figures and Tables

**Table I.** Characteristics, recommended dosage and adverse effects of intravesical drugs.

Drugs	Action site or action period	Recommend dosage and usage	Toxic and side effects	(Refs.)
N,N',N''-	DNA	40-60 mg dissolved in 60 ml of	Bone marrow suppression,	(4,8)
Triethylene	(S designated	normal saline was injected into	digestive tract reaction, including	
thiophosphoramidite	time)	the bladder once a week for a	nausea and vomiting	
		total of 6–8 times		
Gemcitabine	DNA	1 g/50 ml of saline is usually	Bladder irritation reaction,	(10,19,20)
	(S designated	applied once weekly for 6	including frequency, urgency,	
	time)	weeks, or as a single infusion	difficulty in urination, hematuria,	
		immediately after transurethral	bladder or pelvic pain and	
		bladder tumor resection	prostatitis	
Epirubicin	DNA	A single infusion of 80 mg	Cardiac toxicity, gastrointestinal	(17,23)
	(S designated	epirubicin within 6 h from	reaction	
	time)	surgery. 30 mg Epirubicin can		
		also be applied 1–2 weeks after		
		surgery, once a week, for a		
		total of 8 times per course of		
		treatment		
Pirarubicin	G2/M	50 mg immediately after	Bladder stimulation response	(33,35)
	phase	surgery. Generally, the bladder		
		perfusion of 30–40 mg		
		(according to the patient's		
		body surface area) starts from		
		1 week after surgery, once a		
		week, for a total of 8 times		
Mitomycin C	Non-specific	20-40 mg + 30 ml 0.9%	Bladder irritation response, bone	(40,41,43)
	drug with an	sodium chloride injection,	marrow suppression, liver and	
	unclear target	patient position changed every	kidney damage	
		0.5 h		
Bacillus Calmette-	Integrin	The common amount is 120	Bladder stimulation response	(47,63)
Guerin	receptors,	mg, dissolved in 40–50 ml		
	and Toll-like	normal saline and fully		
	receptor 7	shaken		
Interferon	SLC1A1	Giving interferon bladder	Bone marrow suppression,	(64,68)
	solute	perfusion alone requires a	immune response (such as	
	carrier	large dose, generally ranging	headache and fever), hepatorenal	
	family 1	from 5 million units to	toxicity, central nervous system	
	member 1	10 million units	toxicity	
Hemocyanin	Cell-cycle	Not clear	Not clear	(74)
	nonspecific			
	drugs			
Sapylin (Group A	The	Routine perfusion of sapylin 5	Bladder irritation reaction, bone	(75,79,80)
streptococcus for	immune	KE started 1 week after	marrow suppression, and allergic	
injection)	system,	surgery, and intravesical	reaction	
	including	perfusion was retained for 2 h		
	various	once a week for 6 weeks, and		
	immune cells	then once a month for		
	and cytokines	8 months		
Adriamycin	DNA and	Doses were 30–100 mg and	Myelosuppression, cardiotoxicity	(81,82,87)
	mRNA	diluted with saline with a		
		concentration range of		
		0.5–2 mg/ml		

**Table II.** Combined perfusion drugs and effects.

Drugs	Functions	(Refs.)
Bacillus Calmette-Guerin	Reduces recurrence and prevents, or at least delays, the progression of malignancy	(94)
Lidocaine	Effectively improves bladder irritation signs and patient experience	(97)
Antibiotics	Reduces the metabolic loss of antibiotics and reduces side effects	(98)

## Data Availability

The data generated in the present study may be requested from the corresponding author.
